# *QuickStats:* Percentage* of Adults Aged ≥20 Years Who Had Chronic Pain,^†^ by Veteran Status and Age Group — National Health Interview Survey, United States, 2019^§^

**DOI:** 10.15585/mmwr.mm6947a6

**Published:** 2020-11-27

**Figure:**
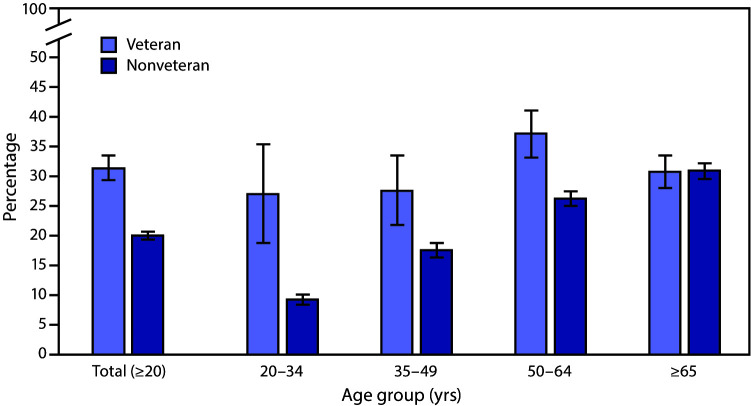
During 2019, military veterans aged ≥20 years were more likely to have chronic pain than were nonveterans (31.5% versus 20.1%). By age group, the likelihood of having chronic pain was higher among veterans than nonveterans for those aged 20–34 years (27.1% versus 9.4%), 35–49 years (27.7% versus 17.7%), and 50–64 years (37.2% versus 26.3%). Among those aged ≥65 years, prevalence of chronic pain did not differ significantly by veteran status (30.8% among veterans versus 31.0% among nonveterans). Among nonveterans, the prevalence of chronic pain increased with age. Among veterans, those aged 50–64 years had the highest prevalence of chronic pain.

**Source:** National Health Interview Survey, 2019. https://www.cdc.gov/nchs/nhis.htm.

For more information on this topic, CDC recommends the following link: https://www.cdc.gov/drugoverdose/prescribing/guideline.html.

